# Drug Hypersensitivity due to Azathioprine with Elevated Procalcitonin

**DOI:** 10.1155/2018/2648325

**Published:** 2018-06-10

**Authors:** Tania Ahuja, Frank R. Chung, Tania Ruiz-Maya

**Affiliations:** ^1^New York University Langone Health, Department of Pharmacy, 550 First Avenue, New York, NY 10016, USA; ^2^New York University School of Medicine, 550 First Avenue, New York, NY 10016, USA; ^3^New York University Langone Health, Department of Medicine, 550 First Avenue, New York, NY 10016, USA

## Abstract

We present a case of azathioprine hypersensitivity presenting as sepsis with elevated procalcitonin in a 68-year-old man with myasthenia gravis. The patient presented with fever, chills, nausea, vomiting, and headache. He developed numerous 1 cm erythematous papules over his upper torso. Infectious workup including bacteriological tests and microbial cultures was negative and a skin biopsy was performed which revealed suppurative folliculitis with eosinophils, consistent with drug hypersensitivity. Notably, acute phase reactants including C-reactive protein and procalcitonin were elevated upon presentation, likely secondary to drug hypersensitivity.

## 1. Background

Azathioprine is an immunosuppressant used in the treatment of various autoimmune conditions, including myasthenia gravis. Azathioprine immune hypersensitivity reaction is a rare adverse effect that has been reported in the literature relatively infrequently; however, the clinical presentation can often mimic sepsis [1, 2]. A review of case reports suggests that azathioprine-induced hypersensitivity reactions most commonly present with fever, chills, rigors, and/or gastrointestinal symptoms [1–7]. Clinicians should become familiar with the clinical presentation to recognize the symptoms and avoid early misdiagnosis.

## 2. Case Presentation

A 68-year-old man who was diagnosed with myasthenia gravis three months prior to admission presented with acute nonpruritic painless 1 cm erythematous papules over the upper torso, accompanied with subjective fevers, chills, nausea, vomiting, and frontal headache for 2 days. His past medical history was significant for heart failure with preserved ejection fraction of 65% and mechanical mitral valve replacement for which he was on warfarin. He was started on prednisone 40 mg daily and pyridostigmine 120 mg four times daily, two and a half months prior to admission, and azathioprine 150 mg daily, 10 days prior to admission. Upon presentation, he was found to have a temperature of 102.7 degrees Fahrenheit, with a heart rate of 107 beats per minute, blood pressure of 159/87 mmHg, and oxygen saturation of 95% on room air.

**Figure 1:**
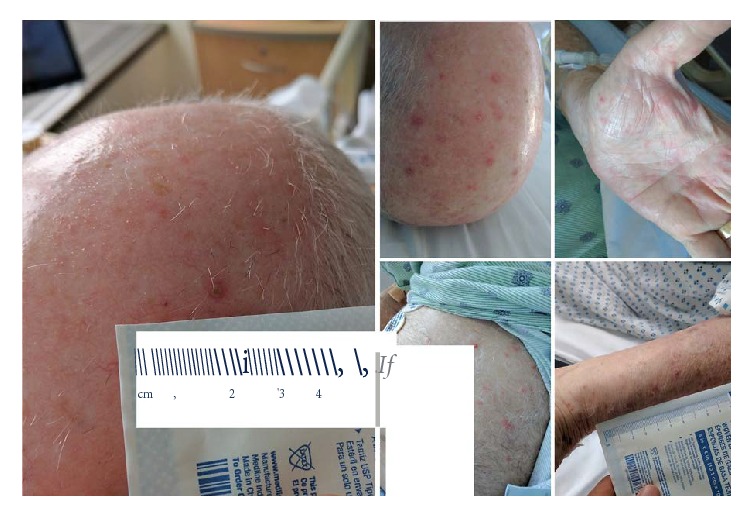
Clinical presentation: firm, indurated, erythematous papules, and nodules, with central pustules and vesicles on the head and scalp. Indurated papules on the palms of the hand. Erythematous pustules on the abdomen.

A complete blood count with differential was remarkable for a white blood cell count of 15,000 cells/mm^3^, with 89% neutrophils and venous lactate of 2.6 mmol/L. All other laboratory parameters including electrolytes, blood urea nitrogen, creatinine, blood glucose, and liver function tests were within normal limits. Given the fever, leukocytosis, and elevated lactate, the initial concern was for sepsis. Infectious workup included blood cultures, chest X-ray, urinalysis with urine culture, respiratory viral panel, Lyme titers, and procalcitonin. The chest X-ray showed a possible new left lower lobe basilar opacity, procalcitonin was 0.59 ng/mL, and the patient was started on antibiotics with ceftriaxone and azithromycin for suspected lower respiratory tract infection. Of note, his azathioprine was discontinued on presentation, due to concern for continued immunosuppression and possible infection. Two days after presentation, given the improvement in clinical symptoms the azathioprine 150 mg was reinitiated. Within a few hours, he became acutely ill, febrile to 103.7 degrees Fahrenheit and tachycardic to 115 beats per minute, with return of the initial presenting symptoms and new onset photophobia. Initially, there was concern for worsening sepsis; repeat procalcitonin was ordered along with C-reactive protein and erythrocyte sedimentation rate (ESR), with antimicrobial therapy broadened to vancomycin, piperacillin/tazobactam, and intravenous acyclovir. Notably, a diffuse 1 cm papulopustular rash erupted over the scalp, head, neck, thorax, abdomen, and upper and lower extremities including the palmar and dorsal aspects of the hand (Figure 1). As the cutaneous findings were nonspecific, the differential remained broad and infectious workup included bacterial, fungal, viral, or drug hypersensitivity. Drug hypersensitivity was suspected given the return of symptoms along with rash after rechallenge of azathioprine and the temporal response to the symptoms. The repeat procalcitonin was now elevated further to 5.36 ng/mL along with an elevated C-reactive protein of >270 mg/L and an ESR of 44 mm/hr.

**Figure 2:**
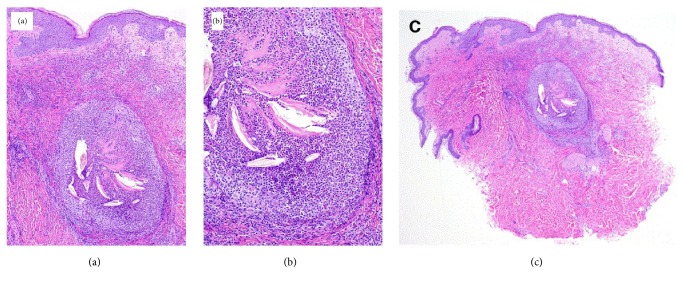
Histopathological features. (a) Lesion of acute ruptured suppurative folliculitis. Papillary dermal edema and neutrophilic dermal infiltration. (b) Dense netrophilic infiltrate in the follicle. (c) Lesion of acute suppurative folliculitis.

The azathioprine was discontinued and the symptoms subsided with the pustules reduced in size and number. Biopsy of the pustule showed suppurative folliculitis, which is expected from a neutrophil driven process, consistent with azathioprine hypersensitivity (Figure 2). All pustule stains, bacterial, viral, including herpes zoster and varicella zoster, and periodic acid-Schiff-diastase (PAS-D) stains, were negative. Repeat liver function tests including AST/ALT remained within normal limits, and a complete blood count revealed a white blood cell count of 9,300 cells/mm^3^ with 0% eosinophils. Antimicrobial therapy was deescalated. Over the next few days, the rash and symptoms resolved and the CRP decreased to 108 g/L. We utilized the Naranjo algorithm to estimate the probability of azathioprine causing hypersensitivity and found that our patient had a probable hypersensitivity reaction to azathioprine [8].

## 3. Discussion

Drug hypersensitivity syndrome (DHS) is often reported in patients treated with aromatic antiepileptic drugs and in rare cases with azathioprine [9]. The predominant cutaneous reaction reported in the literature is a neutrophilic dermatosis [9]. Other cutaneous effects may include an erythematous macular or maculopapular eruption and purpuric or petechial lesions [2, 7]. Azathioprine inhibits DNA and RNA synthesis and is used in a multitude of conditions from inflammatory bowel disease to autoimmune diseases such as connective tissue disease, rheumatoid arthritis, and myasthenia gravis amongst other things. It is a purine antagonist that is metabolized by thiopurine methyltransferase (TPMT) to the active compound, 6-mercaptopurine; however this metabolism seems to be unrelated to the incidence of hypersensitivity [10]. Furthermore, azathioprine has an inactive metabolite, methyl nitroimidazole, which may be responsible for the hypersensitivity reactions due to the generation of a hapten during metabolism [4, 9, 11, 12]. Although rare, there have been about 70 cases of azathioprine-induced hypersensitivity reported in the literature thus far, with concurrent corticosteroids at presentation being present in 39% of patients [9]. The onset of symptoms presented anywhere from 3-4 days of initiation of azathioprine, with 49% of patients exhibiting cutaneous findings suggesting a drug-induced acneiform and folliculitis eruption [9]. Although the systemic symptoms of azathioprine hypersensitivity include fever, chills, arthralgias, myalgias, leukocytosis, and cutaneous eruptions, other laboratory parameters may be unremarkable [1, 9]. Of note, the possibility of disseminated herpes or zoster should be considered in immunosuppressed patients hospitalized with acute folliculitis. It is critical to obtain a skin swab from the base of an intact unproofed pustule to submit for HSV and VZV PCR, as was done in our case, despite the pustular morphology. In addition, a bedside Tzanck smear is a rapid test and the dermatopathologist may find evidence of viral cytopathic changes. These findings were not found in our patient.

On initial presentation, azathioprine hypersensitivity may be confused with infection, as was the case in our patient. We observed an elevated procalcitonin in our patient, which has been identified as a biomarker to differentiate bacterial infections from viral infections and noninfectious inflammatory conditions [13]. For sepsis, procalcitonin, along with other diagnostic tools and physical examination, has been found to be more sensitive and more specific than other biomarkers, including CRP and lactate [13, 14]. Therefore, procalcitonin is often checked in patients that present with a sepsis like picture at our institution in the emergency room. However, elevations in acute phase reactants, such as procalcitonin, may lead to misdiagnosis and delay in management of drug related hypersensitivity reactions. In one retrospective review of drug reactions with eosinophilia and systemic symptoms, procalcitonin was found to be elevated to levels normally seen in bacterial infections or sepsis [15]. Similarly, we found that procalcitonin levels were elevated alongside other acute phase reactants, despite having a nonbacterial etiology and suspected drug hypersensitivity. This suggests that an elevated procalcitonin must be interpreted with caution in patients who may have proinflammatory etiologies beyond infections, such as hypersensitivity reactions, as this may result in anchoring and delay prompt diagnosis of the true etiology. While further studies are needed to determine the relationship between procalcitonin and drug hypersensitivity, this report illustrates the need for clinical suspicion for a drug-induced hypersensitivity syndrome in patients started on azathioprine presenting with a sepsis like syndrome.

Other acute phase reactants that may be elevated with drug hypersensitivity include C-reactive protein and ESR, as was the case in our patient. Further, azathioprine hypersensitivity is often suspected upon resuming or rechallenge with azathioprine, where symptoms may recur within hours of the dose [16], consistent with our patient. Although he was not prescribed azathioprine on admission to the hospital, given the suspicion for sepsis, azathioprine was reinitiated in the hospital with return of symptoms. The timing of presentation with azathioprine hypersensitivity is critical to the diagnosis to distinguish from other idiosyncratic drug reactions such as DRESS (Drug Reaction with Eosinophilia and Systemic Symptom) [16]. Patients often present within 4 weeks of initiation of azathioprine and symptoms resolve within 7 days of drug cessation, with symptoms returning within hours of rechallenge [16], as was seen in our case. Given the severity of the reaction, patients that experience fever, hypotension, or a severe reaction should not be rechallenged [1, 16, 17].

In conclusion, we report a case of azathioprine hypersensitivity with cutaneous manifestations of neutrophilic dermatoses along with elevated serum CRP and procalcitonin. Early recognition of the signs and symptoms of azathioprine hypersensitivity may help clinicians in detecting and preventing further sequelae. Further analyses are required to determine the correlation of these acute phase reactants with the severity and outcome of hypersensitivity to medications.
